# T/R RF Switch with 150 ns Switching Time and over 100 dBc IMD for Wideband Mobile Applications in Thick Oxide SOI Process

**DOI:** 10.3390/s22020507

**Published:** 2022-01-10

**Authors:** Behnam S. Rikan, David Kim, Kyung-Duk Choi, Arash Hejazi, Joon-Mo Yoo, YoungGun Pu, Seokkee Kim, Hyungki Huh, Yeonjae Jung, Kang-Yoon Lee

**Affiliations:** 1 Department of Electrical and Computer Engineering, Sungkyunkwan University, Suwon 16419, Korea; behnam@skku.edu (B.S.R.); dkim9402@skku.edu (D.K.); glyiiop@skku.edu (K.-D.C.); arash@skku.edu (A.H.); fiance2@g.skku.edu (J.-M.Y.); hara1015@skku.edu (Y.P.); seokkeekim@skku.edu (S.K.); gray.huh@skku.edu (H.H.); yj.jung@skku.edu (Y.J.); 2 SKAIChips Co., Ltd., Suwon 16419, Korea

**Keywords:** fast switching time, inter-modulation distortion, silicon-on-insulator, single pole double throw switch, T/R RF switch

## Abstract

This paper presents a fast-switching Transmit/Receive (T/R) Single-Pole-Double-Throw (SPDT) Radio Frequency (RF) switch. Thorough analyses have been conducted to choose the optimum number of stacks, transistor sizes, gate and body voltages, to satisfy the required specifications. This switch applies six stacks of series and shunt transistors as big as 3.9 mm/160 nm and 0.75 mm/160 nm, respectively. A negative charge pump and a voltage booster generate the negative and boosted control voltages to improve the harmonics and to keep Inter-Modulation Distortion (IMD) performance of the switch over 100 dBc. A Low Drop-Out (LDO) regulator limits the boosted voltage in Absolute Maximum Rating (AMR) conditions and improves the switch performance for Process, Voltage and Temperature (PVT) variations. To reduce the size, a dense custom-made capacitor consisting of different types of capacitors has been presented where they have been placed over each other in layout considering the Design Rule Checks (DRC) and applied in negative charge pump, voltage booster and LDO. This switch has been fabricated and tested in a 90 nm Silicon-on-Insulator (SOI) process. The second and third IMD for all specified blockers remain over 100 dBc and the switching time as fast as 150 ns has been achieved. The Insertion Loss (IL) and isolation at 2.7 GHz are −0.17 dB and −33 dB, respectively. This design consumes 145 uA from supply voltage range of 1.65 V to 1.95 V and occupies 440 × 472 µm^2^ of die area.

## 1. Introduction

The rapid growth of wireless communication standards at different frequency bands such as Global Systems for Mobile communications (GSM), Code-Division Multiple Access (CDMA) and the expansion of other non-cellular wireless services, such as WiFi, Bluetooth, and Global Positioning System (GPS), place the Radio Frequency (RF) switches in an increasingly vital role in the RF front-end module for mobile terminals [1].

In Time-Division Duplexing (TDD) systems, when the signal transmission and reception happens at different time slots, a circulator is employed in remote antenna unit to provide the isolation between the Tx and Rx connected to the same antenna. The circulator is costly while the antenna switch can be integrated into the same chip.

Conventional structure of the RF switches includes the switch core and driving circuitry (negative charge pump and voltage generator) as presented in [2,3,4]. The work proposed in [5] employs a new biasing scheme and almost shows the same performance metrics with negative biasing architecture. However, there is a significant gap in isolation performance between the measurement and simulated result due to leakage of RF signal from ON branch to OFF branch through the biasing intermediate nodes.

Since the target output power of the Tx is 30 dBm, improving the power handling capability and short switching time are the main improvement strategies. As the power increases, the isolation performance becomes more critical; therefore, the returned RF signal to the analog part must be significantly attenuated to improve the isolation and any breakdown in analog parts. Furthermore, Inter-Modulation Distortion (IMD) is another important parameter in RF switch design, but it is usually not being reported in papers. We have presented thorough analysis of this parameter.

One of the attractive processes for RF switch applications is GaAs pHEMT [6,7,8,9,10,11] which dissipates low power, and has low insertion loss (IL), and high power-handling characteristics. Nevertheless, Silicon-on-Insulator (SOI) has become dominant in the design of RF switches [12,13,14,15,16,17,18,19,20,21] recently, due to its capabilities to operate and fabricate at a low supply voltage, as well as to integrate Complementary Metal–Oxide–Semiconductor (CMOS) control logic circuits on chip.

In this paper, we discuss a low power, fast switching time Single Pole Double Throw (SPDT) RF switch design in a thick SOI process. We discuss and analyze the switch core design considerations such as size of transistors, number of stacks and gate/body control voltages thoroughly. Furthermore, the analog perimeter circuitry applied in the design has been presented.

The remainder of this paper is organized as follows: Section 2 discusses the low power SPDT switch design considerations. In Section 3, we have presented analog control circuitry. Experimental results are summarized in Section 4, and finally Section 5 concludes the paper.

## 2. Low-Power SPDT Switch Design Considerations

**Figure 1:**
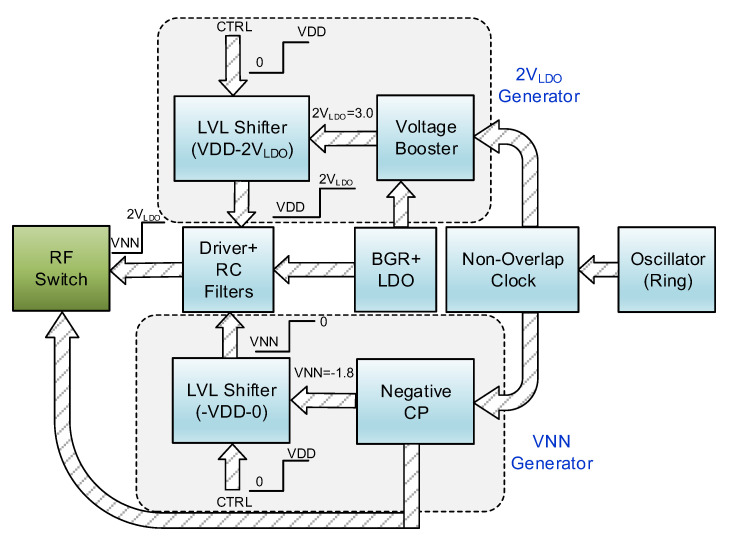
Block diagram of the designed SPDT RF switch and analog circuitry.

Figure 1 shows the block diagram of the designed SPDT RF switch and the analog circuitry to control the switch. It includes a ring oscillator to generate clock and a non-overlap clock generator to generate the control clocks for voltage booster and negative charge pump. The negative charge pump and voltage booster generate VNN (which is −VDD) and VBB (which is +2V_LDO_) voltages to be applied in the circuit. Level shifters shift the control signals from 0–VDD range to VNN–0 and VDD–2V_LDO_ ranges and drivers generate VNN–2V_LDO_ control signals to the RF switches. A Low Drop-out Regulator (LDO), which includes a Bandgap Reference (BGR), generates a stable 1.5 V voltage for the system. We discuss the circuitry of each of these sub-blocks in the next section.

**Figure 2:**
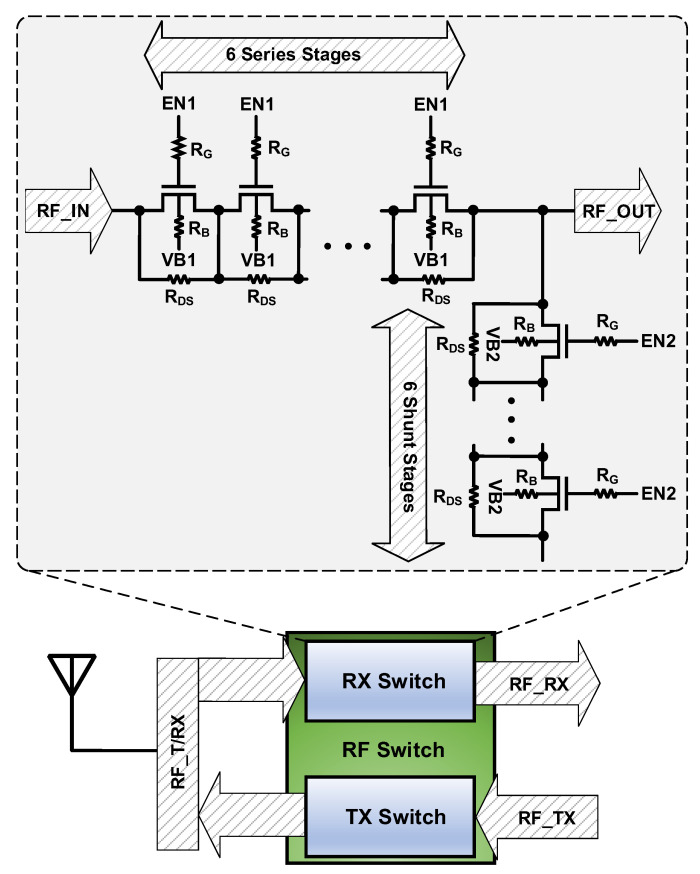
Schematic of the designed SPDT T/R RF switch.

In the design of the RF switches, the fundamental specifications to be considered are insertion loss and isolation. Furthermore, power handling, linearity and harmonic rejections are also the issues that should be considered. For this design, the switching time is also considered as it has fast switching specification. Figure 2 presents the schematic of the designed SPDT T/R RF switch. It includes six transistors stacks as series and six transistors stacks as shunt parts. R_G_ and R_B_ resistors isolate the bias circuits of the gate and body from RF signal. R_DS_ has been placed between source and drain of the transistors to present dc voltage potential from developing in the stack. For this design a 90 nm RSB technology has been applied where we have used 2.5 V thick oxide RF transistors in our switch design.

### 2.1. Insertion Loss

**Figure 3:**
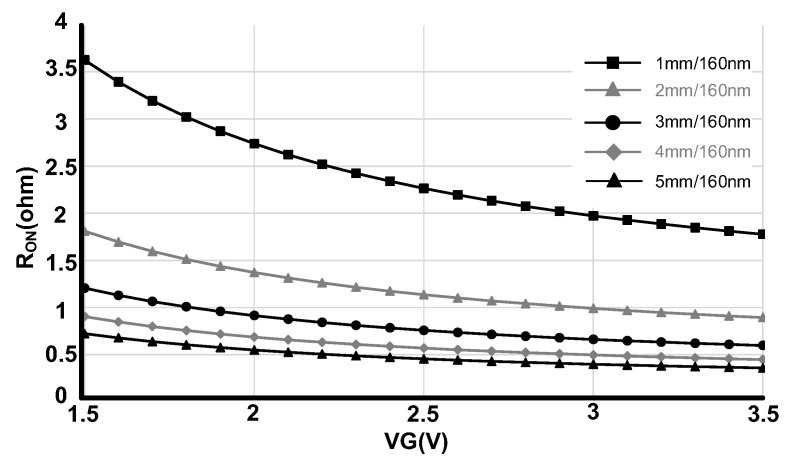
R_ON_ of the transistor with respect to the size and gate voltages.

The insertion loss is directly proportional to the R_ON_ of the transistors. Although, the body-contacted transistors show higher R_ON_ in comparison with floated body transistors, we have applied these transistors due to better harmonic performances [1]. To improve the R_ON_ and consequently insertion loss specifications, the size of each series transistor has been selected as big as 3.9 mm/160 nm. Furthermore, a boosted bias voltage has been applied to the gate of the transistors while the body has been connected to 0 V. Figure 3 shows the simulated R_ON_ of the transistor with respect to the size and applied gate voltages.

While applying gate voltage, we should consider the gate-oxide breakdown and Absolute Maximum Rating (AMR) conditions as well. The AMR condition specifies that the supply voltage can rise up to 2.5 V. Boosting this voltage would increase the control signal of the gate up to 5 V, which has less margin with the gate-oxide breakdown specifications (5.4 V) of the applied transistors; therefore, an LDO has been applied where it has a regulated 1.5 V output voltage for all ranges of the supply voltages from 1.65 (Minimum supply voltage) to 2.5 V (AMR conditions). With 1.5 V output voltage of the LDO as the supply voltage of the voltage booster, the boosted voltage will not go over 3 V, which satisfies the gate-oxide breakdown voltage of the transistors with enough margin.

Referring to the R_ON_ values on Figure 3, each stack of the transistors in series path has almost 0.5 Ohm for 3 V gate voltage and 3.9 mm/160 nm transistor sizes. This value for the shunt transistors where the size is 0.75 mm/160 nm and the gate voltage is 3 V is about 2.6 Ohms. The shunt transistors connect the output port of the OFF stage to the ground.

### 2.2. Isolation

**Figure 4:**
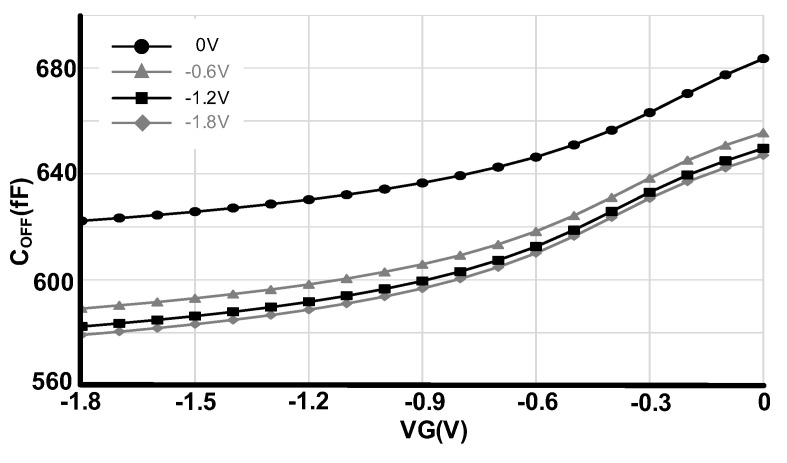
C_OFF_ for different gate and body voltages.

The isolation of the RF switch is determined by how well the transistor has been turned off and this is determined by the off-state capacitance (C_OFF_). Figure 4 shows the simulated OFF-state capacitance for different gate and body voltages from 0 to −1.8 V. The gate voltage has been swept from −1.8 V to 0 V for 0 V, −0.6 V, −1.2 V and −1.8 V body voltages. Unlike the voltage booster, the negative charge pump has been directly connected to supply voltage to provide lower negative values and consequently better isolation. As we can observe from Figure 4, the lowest C_OFF_ capacitance happens at VG = −1.8 V and VB = −1.8 V. Therefore, for the OFF case and to improve the isolation, we have applied −1.8 V to the gate and −1.8 V to the bulk of the transistors.

**Figure 5:**
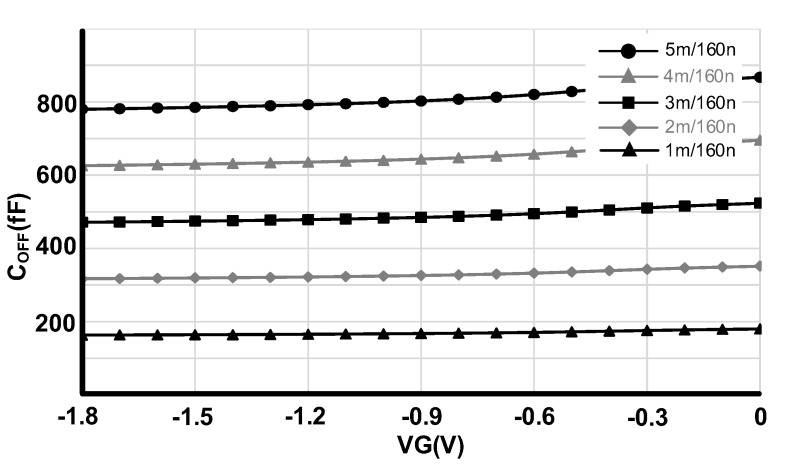
C_OFF_ with respect to transistor size and gate voltages.

C_OFF_ has also relation with switch transistors size. As it has been shown on Figure 5. the bigger the size of the transistor, the C_OFF_ of the switch increases, therefore degrading the isolation. This is in contradiction with the R_ON_ and insertion loss analysis where the size increment improved those specifications. Increasing the width of the transistors improves the insertion loss in the ON state while degrades the isolation in the OFF state; therefore, an optimum value should be selected to satisfy both specifications. Further, the IMD2 and IMD3 specifications have relation with the transistor sizes, which is discussed in the next sections of this paper.

### 2.3. Power Handling

**Figure 6:**
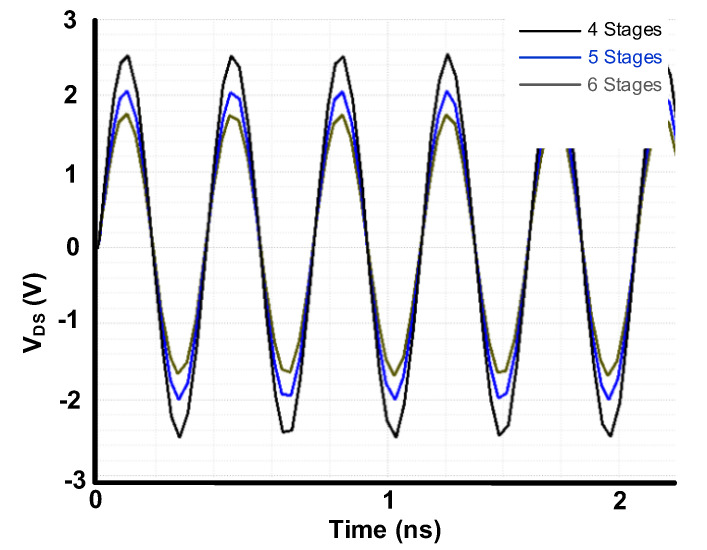
V_DS_ of first transistor vs. number of stacks in series for OFF cases.

One of the stringent specifications to be satisfied in RF switch design is power handling capability. This is especially more critical in high-power applications such as GSM. This specification is the capability of the switch in handling high powers without breaking down the transistors. The power handling capability is improved by stacking the transistors in the switch. In the stacks of transistors, the first transistor has always the most stress and would breakdown first. In Figure 6, we have plotted the V_DS_ of first transistor vs. number of stacks in series for OFF cases as an example and with 30 dBm power which is the AMR condition for this design. As it can be seen from Figure 6, increasing the number of stacks reduces the V_DS_ and consequently improves the power handling capability of the switch. For this design, considering the power handling specifications, we have applied six stacks of transistors for both series and shunt parts.

### 2.4. Harmonics

**Figure 7:**
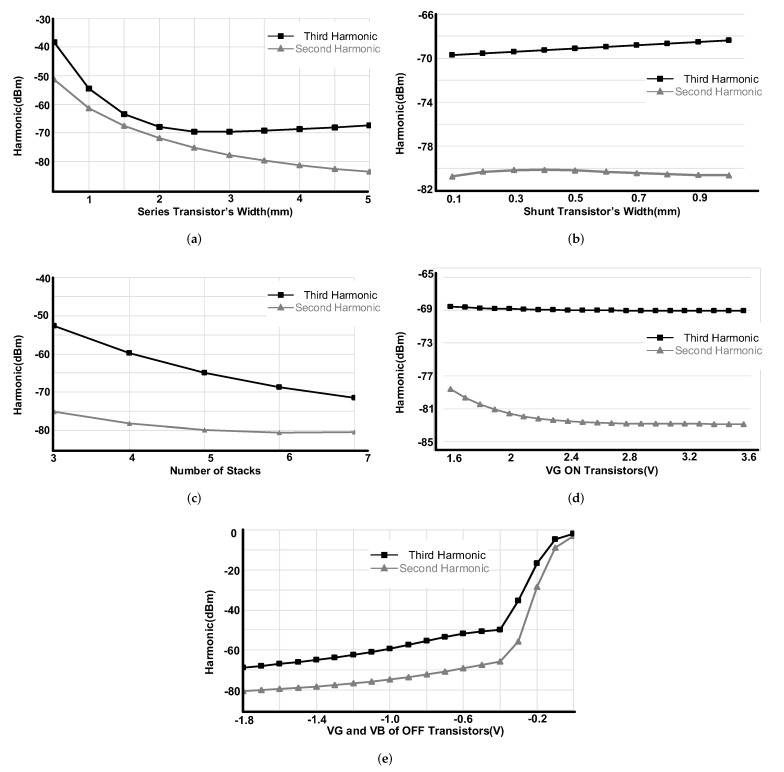
H2 and H3 variation vs. (**a**) series transistors width, (**b**) shunt transistors width, (**c**) number of stacks, (**d**) ON transistors gate voltage and (**e**) OFF transistors gate and body voltage.

The current limitations in ON stage and the voltage limitation in OFF stage decide the harmonic distortions performance of the switch. These limitations are decided according to the number of the stages and size of the transistors. Figure 7a,b show the second and third harmonic performance vs. series and shunt transistors size swept for six stacks of the transistors. As it is shown on Figure 7a, increasing the transistor sizes up to 5 mm improves second harmonic. Nevertheless, for the third harmonic, the performance over 2 mm is almost constant.

Figure 7c shows the harmonic performance vs. number of stacks. Both second and third harmonics improve by increasing the number of stacks (the insertion loss degrades with number of stacks as total R_ON_ increases). For these simulations the gate and body of the ON transistors have been connected to 1.8 V and 0 V, respectively, while both of these values for OFF transistors are −1.8 V.

Figure 7d,e show the harmonic performance vs. different gate and body voltages. In Figure 7d the control voltage of the ON transistors have been swept from 1.6 V to 3.6 V while the OFF-transistors body and gate have been connected to −1.8 V. The second harmonic shows improvement by gate control voltage increment. In Figure 7e the ON transistors gate voltage have been connected to 1.8 V and the gate and body of the OFF transistors have been swept from −1.8 V to 0 V. For simulations in Figure 7c–e the size of the series and shunt transistors have been selected 3.9 mm/160 nm and 0.75 mm/160 nm, respectively. It is important to note that these analyses are for this specific 90 nm RSB PDK where for other PDKs the variation and tendency can be slightly different. Furthermore, the summarized results are for the core switch excluding the effects of the analog control circuitry.

### 2.5. Switching Time

**Figure 8:**
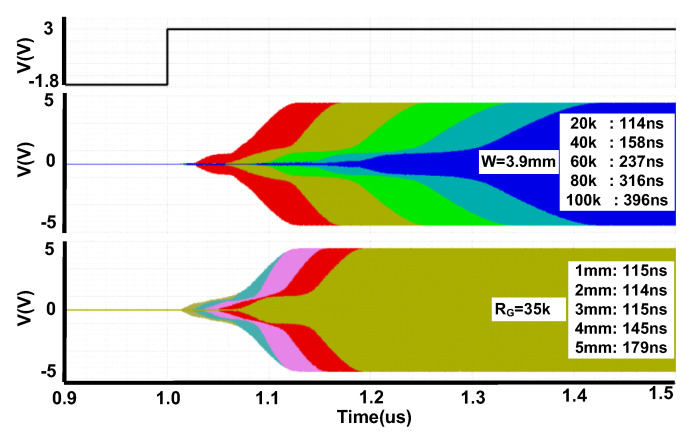
Switching time vs. different width and different gate resistors.

The switching time in RF switches is defined as the time between the state where the RF input and control signals switch to active mode and time where the signal at the other port achieves 90% of the RF signal. This time is critical in some of the RF switch designs (as for the design in this paper where the defined specification forces us to keep this value below 300 ns). The number of stacks, gate resistor size and transistors size are the most important factors that decide switching time of the switches. Switching time vs. different width and also different gate resistors have been summarized in Figure 8.

All of the above analysis and results have been performed in 2.7 GHz frequency. Decision of the number of the stacks and optimized sizes for transistors depends on the start-up time, harmonics, insertion loss and IMD performance specifications. According to the above analysis, we have selected the size of each shunt transistor 750 um/160 nm and each of the series transistor 3.9 mm/160 nm. To improve the switching time, the gate resistors have been selected to be as small as 35 kOhms. There are also 150 kOhms resistors between drain and source of each transistor and also in the body of each transistor. The gate and body of the transistors in the ON stage have been biased with boosted 3 V and 0 V, respectively. This improves the harmonic performance as well as the R_ON_ of the transistors, which consequently improves the insertion loss. The gate and body of the transistors in the OFF stage have been biased with −1.8 V and −1.8 V, respectively. This also improves the harmonic performance and isolation as well.

## 3. Analog Control Circuitry

In this section we briefly review the analog circuits that have been applied in this design to control the RF switch.

### 3.1. BGR and LDO

**Figure 9:**
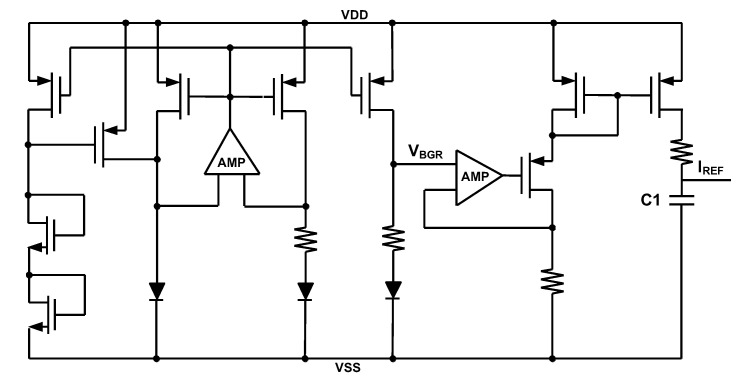
BGR and the reference current generator.

**Figure 10:**
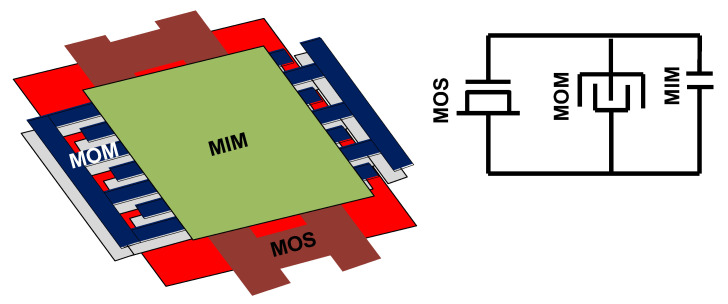
Custom-made capacitor made of MOS, MOM and MIM capacitors.

**Figure 11:**
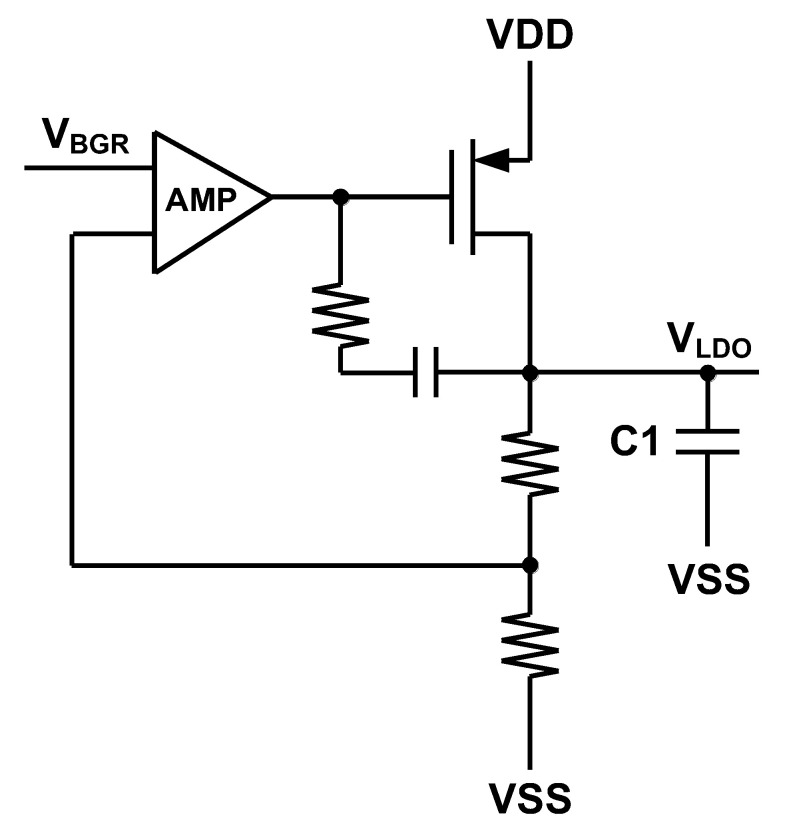
LDO schematic.

Figure 9 shows the structure of the designed Bandgap Reference (BGR) and the reference current generator. V_BGR_ is used in LDO and I_REF_ has been applied in ring oscillator. As there are no Bipolar Junction Transistors (BJT) in the applied process, we have applied diodes instead of BJTs to design the BGR. In order to reduce the area of analog parts, which is dominated by capacitors in BGR, Negative Charge Pump (NCP), etc., a custom-made capacitor has been made. This is a dense custom-made capacitor made of a Metal-Oxide-Semiconductor (MOS) capacitor, a Metal-Oxide-Metal (MOM) capacitor and a Metal-Insulator-Metal (MIM) capacitor where following Design Rule Check (DRC), all of these capacitors have been placed over each other in layout as it is shown in Figure 10. The structure of the LDO has been shown in Figure 11 [22,23]. In this structure also the custom-made capacitors have been applied at the output of the LDO. V_LDO_ is almost 1.5 V and has been applied to the voltage booster, level-shifters and drivers but not the negative charge pump. The reason not to apply the LDO voltage to the negative charge pump is to obtain more negative values. The AMR conditions are also satisfied even if lower voltages are applied to the gate and body of the switches.

### 3.2. Non-Overlap Clock Generator

**Figure 12:**
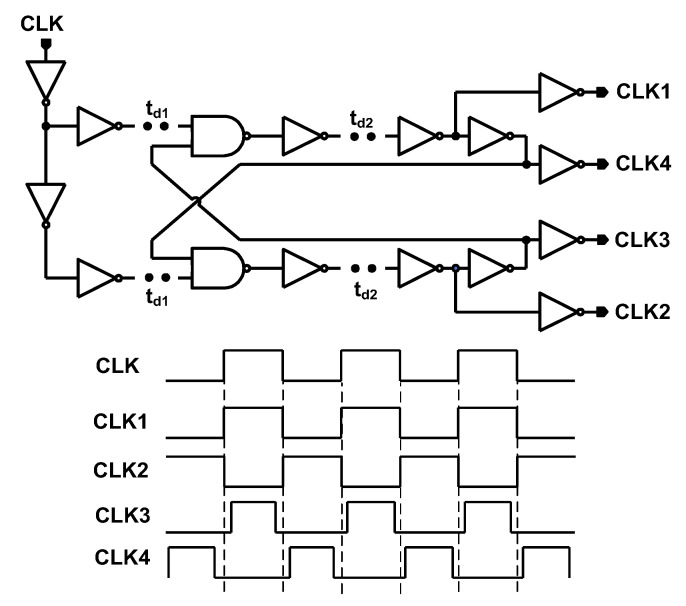
Non-overlap clock generator.

The structure of the non-overlap clock generator is presented in Figure 12 [24]. The generated non-overlap clocks (CLK1-CLK4) have been applied in voltage booster and negative charge pump.

### 3.3. Negative Charge Pump and Voltage Booster

**Figure 13:**
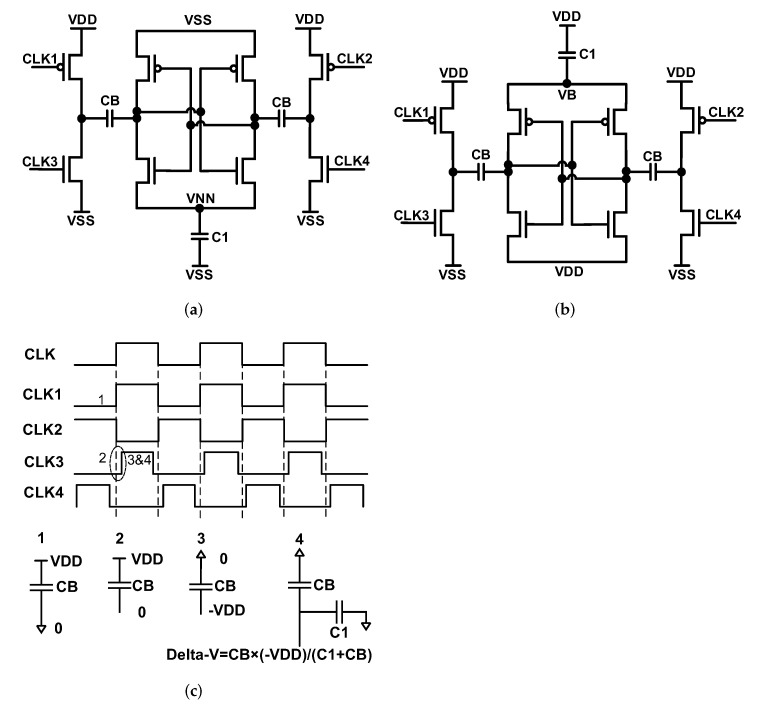
(**a**) Negative charge pump, (**b**) voltage booster and (**c**) process of charging and storing on storing capacitor.

Structure of the applied negative charge pump and voltage booster have been presented in Figure 13a,b, respectively [25,26]. The non-overlap clocks control the charging and storing procedure of these structures. The process of charging and storing the charge on the storing capacitor (C1) has been summarized in Figure 13c for NCP. For voltage booster the procedure is the same. On each clock cycle, each of the CB capacitors charges and stores on the storing capacitance once; one in the rising edge of the clock and the other one in the falling edge of the clock.

The decision of the size of the C1 capacitor depends on loads that are the RF switches and drivers. The size of this capacitor largely decides the size of the analog control parts. Especially, for the fast switching structures, the size of this capacitor needs to be high enough that it does not discharge significantly while there is switching in RF circuitry. That is why we have applied custom-made dense capacitors for this part. The value of CB capacitors are usually several tens of times smaller than C1 capacitor due to size and ripple control. For CB, only MIM capacitors have been applied. The amount of VNN voltage that changes in each charging, is the ratio of the CB and CB + C1 capacitors multiplied by -VDD as it is summarized in Figure 13c.

### 3.4. Level Shifters and Drivers

**Figure 14:**
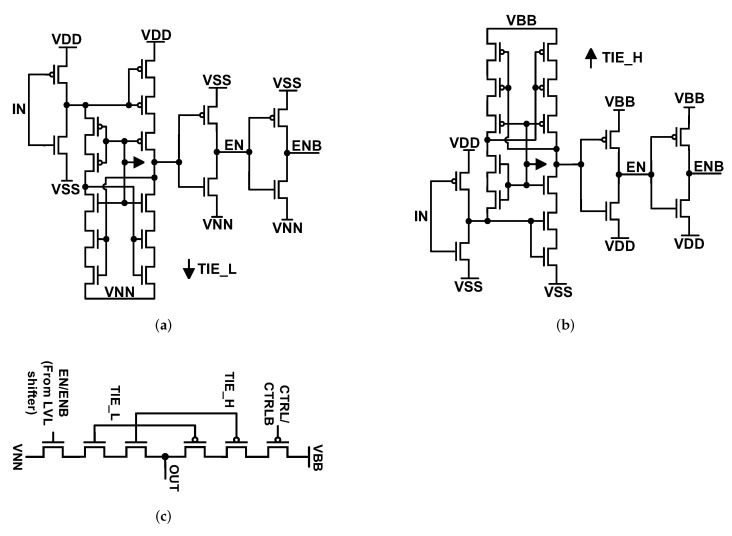
(**a**) Negative level shifter, (**b**) boost level shifter and (**c**) driver structure.

Level shifters that have been applied in this design have been shown in Figure 14a,b [27]. Figure 14a shifts the control signals from 0−VDD range to VNN−0 range where VNN is −VDD. Figure 14b shifts the signal from 0−VDD range to VDD−VBB range where VBB is 2V_LDO_. Due to AMR conditions and to keep V_DS_ of transistors low enough, some transistors have been stacked on these designs. The structure of the applied driver has been presented in Figure 14c. Again, for this design, AMR conditions forced us to stack the transistors and connect them to VDD or VSS to divide the voltages evenly over V_DS_ of transistors.

### 3.5. Ring Oscillator

**Figure 15:**
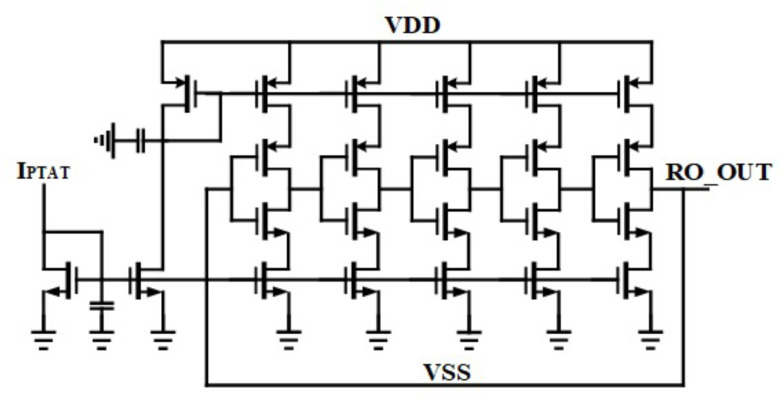
Ring oscillator structure.

Since the architecture of the negative CP does not need accurate frequency generation, the oscillator is implemented as a five-stage ring oscillator (RO) [28]. The delay stages are based on current starved delay cells in which the output frequency is controlled by the current of each branch of the delay cells. The output frequency of the RO is set to 20 MHz at TT corner with the temperature of 25 °C and supply voltage of 1.8 V, while it has a frequency variation of about ±15 MHz considering process, voltage and temperature variations. The effect of the frequency variation of the RO on switching time is negligible. However, it can change the settling time of the negative voltage generation by the NCP. The structure of the designed RO has been summarized in Figure 15.

## 4. Experimental Results

**Figure 16:**
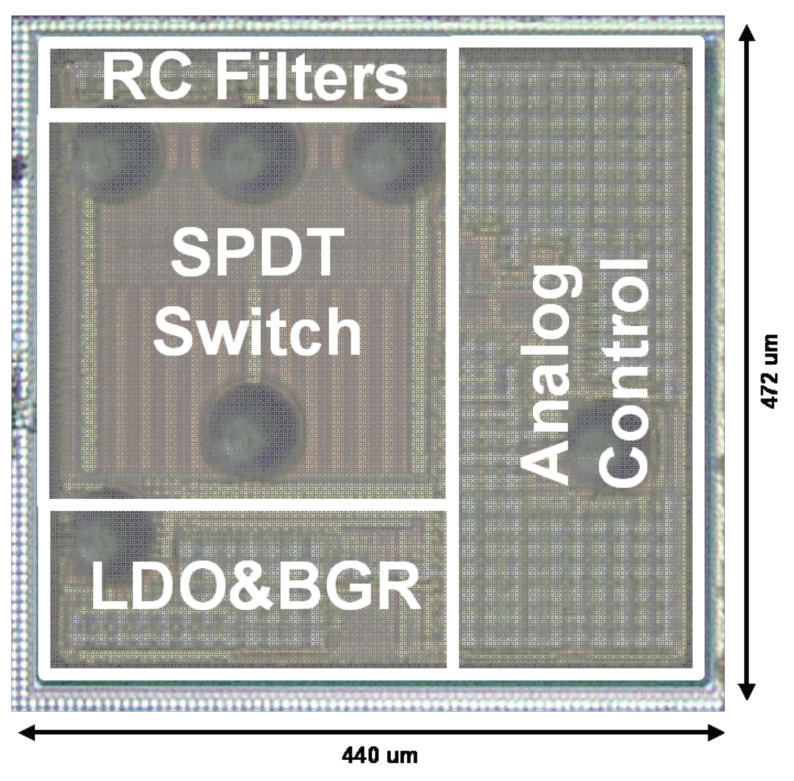
Chip micrograph.

The low-power SPDT has been designed and tested in a 90 nm RSB process design kit. The 2.5 V transistors that have been applied are switch specialized SOI devices. Figure 16 shows the chip micrograph of the designed low-power SPDT. The occupied area for this design is 440 × 472 µm^2^. This switch and the analog control parts consumes 145 µA from a 1.8 V supply voltage. The operation frequency range is 50 MHz–6 GHz.

**Figure 17:**
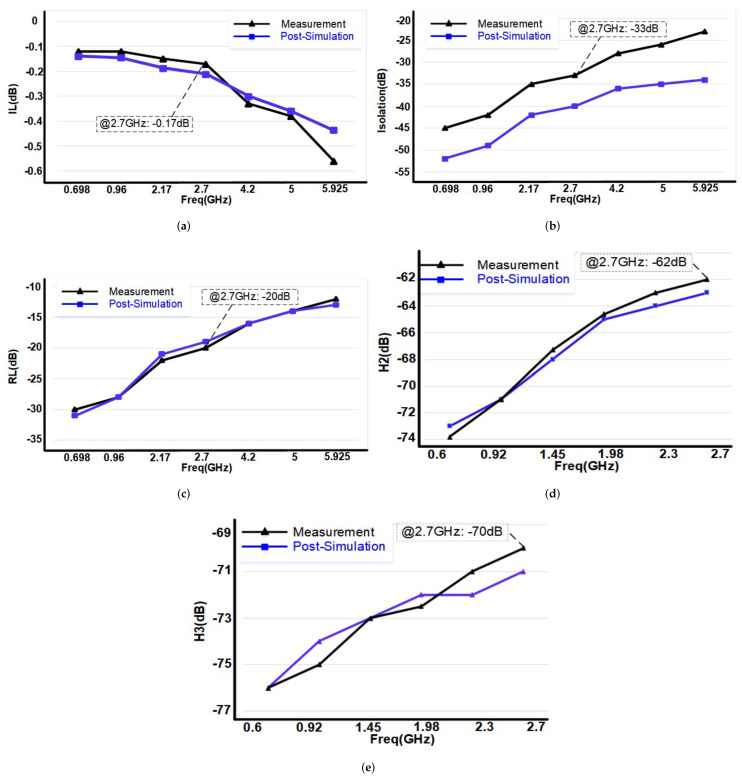
Measured and post-simulated (**a**) insertion loss, (**b**) isolation, (**c**) return loss, (**d**) second harmonic and (**e**) third harmonic.

Both post-simulations and measurements of IL have been summarized in Figure 17a. For the range from 600 MHz to 6 GHz the post-simulated and measured insertion loss values are −0.13∼−0.44 and −0.12∼−0.56 dB, respectively. The post-simulations and measurements of isolation have been summarized in Figure 17b. For the range from 600 MHz to 6 GHz the post-simulated and measured isolation values are −52∼−34 and −45∼−23 dB, respectively. Figure 17c presents the post-simulation and measurement results of the return loss. For the range from 600 MHz to 6 GHz the post-simulated and measured return loss values are −31∼−13 and −30∼−12 dB, respectively.

Figure 17d,e present the post-simulated and measured second and third harmonics performance. For the range from 600 MHz to 2.7 GHz the post-simulated and measured second harmonic values are −73∼−63 and −74∼−62 dB, respectively. For third harmonic, these values are −76∼−71 and −76∼−70 dB, respectively.

Table 1 summarizes the measured IMD2 of the switch in different bands where the blockers power and frequencies have been specified. As we can see, for all the mentioned bands (B1, B7 and B8) and applying different blocker 1 power, the IMD2 < −85 dBm achieved (>109 dBc for worst case). Table 2 summarizes the measured IMD3 of the switch in different bands where the blockers power and frequencies have been specified. As we can see, for all the mentioned bands IMD3 < −100 dBm achieved (>125 dBc for worst case). The measured switching time for this structure is 150 ns.

Table 3 summarizes the performance of the proposed RF switch with recent works and products [4,29,30]. The proposed RF switch features low insertion loss, return loss, and higher isolation over a broad frequency range. Compared to commercial switch product, the presented configuration demonstrates a comparable performance metrics in both high power and low power harmonic and IMD test cases. In addition, a switching time of less than 150 ns is a promising solution for high speed applications. Finally, maximum power handling performance of this design is comparable to the commercial product that has been reported in [30].

## 5. Conclusions

This paper presented a fast-switching T/R SPDT RF switch. Thorough analyses have been presented to choose the optimum number of stacks, transistor sizes, gate and body voltages to satisfy the required specifications. The implemented switch applied six stacks of series and shunt transistors with 3.9 mm/160 nm and 0.75 mm/160 nm sizes, respectively. To satisfy the switching time, 35 kOhms resistors have been applied at the gate of each transistor. A negative charge pump and a voltage booster generate the negative and boosted control voltages to improve the harmonics and to keep intermodulation distortion performance of the switch below −100 dBc for blockers of the different frequencies and different powers. A low drop-out regulator has been applied to limit the boosted voltage to the AMR conditions and to improve the switch performance for process, voltage and temperature variations. To reduce the size, a dense custom-made capacitor consisting of a metal–insulator–metal, a metal–oxide–metal and a metal–oxide–semiconductor capacitors has been presented where they have been placed over each other in layout considering the DRC and applied in negative charge pump, voltage booster and LDO. This switch has been fabricated and tested in a 90 nm SOI process. The IMD2 and IMD3 for all specified blockers remain over 100 dBc and the switching time as fast as 150 ns has been achieved. The IL and isolation at 2.7 GHz are −0.17 dB and −33 dB, respectively. This design consumes 145 µA from supply voltage range of 1.65 V to 1.95 V and occupies 440 × 472 µm^2^ of the die area.

## Figures and Tables

**Table 1:** IMD2 measurement summary.

Band	In-Band Freq.	Blocker Freq.1	Blocker Power1	Blocker Freq.2	Blocker Power2	IMD2
	(MHz)	(MHz)	(dBm)	(MHz)	(dBm)	(dBm)
B1	2140	1950	15	190	−15	−106
B8	942.5	897.5	15	45	−15	−94
B7	2655	2535	15	120	−15	−100
B1	2140	1950	20	190	−15	−101
B8	942.5	897.5	20	45	−15	−89
B7	2655	2535	20	120	−15	−95
B1	2140	1950	24	190	−15	−97
B8	942.5	897.5	24	45	−15	−85
B7	2655	2535	24	120	−15	−91

**Table 2:** IMD3 measurement summary.

Band	In-Band Freq.	Blocker Freq.1	Blocker Power1	Blocker Freq.2	Blocker Power2	IMD3
	(MHz)	(MHz)	(dBm)	(MHz)	(dBm)	(dBm)
B1	2140	1950	15	1760	−15	−124
B8	942.5	897.5	15	852.5	−15	−130
B7	2655	2535	15	2415	−15	−125
B1	2140	1950	20	1760	−15	−114
B8	942.5	897.5	20	852.5	−15	−120
B7	2655	2535	20	2415	−15	−111
B1	2140	1950	24	1760	−15	−106
B8	942.5	897.5	24	852.5	−15	−112
B7	2655	2535	24	2415	−15	−101

**Table 3:** Comparison with recent works and products.

Parameter	TMTT 2015 [5]	TMTT 2008 [29]	ESSCIRC 2010 [4]	Infineon BGS12SN6 [30]	This Work
Architecture	SP4T	SPDT	SP4T	SPDT	SPDT
Frequency (GHz)	1–2	1	1–2	0.05–6	0.698–5.925
Insertion Loss (dB)	0.55–0.75	0.55	0.27–0.34	0.23–0.9	0.12–0.56
Return Loss (dB)	30–20	30	30–24	22–16	30–12
Isolation (dB)	39.4–32	39.4	40–35	43–21	45–23
2nd Harm. (dBc)	82–83	82	90–84	Typ:80, Max:75 ^3^	62 + 24 ^1^
3rd Harm. (dBc)	80–81	80	87–80	Typ:87, Max:80 ^3^	76 + 24 ^1^
IMD2 (dBm)	-	-	-	Typ:110, Max:100 ^2^	Typ:94, Max:106 ^4^
IMD3 (dBm)	-	-	-	Typ:130, Max:120 ^2^	Typ:124, Max:130 ^4^
Supply Voltage (V)	2.5	3.3	2.5	2.85	1.8
Switching Time (µm)	-	-	-	0.4	0.15
Power Handling (dBm)	35	33	35	32	32

^1^ P_in_ = 24 dBm, Frequency = 2.7 GHz; ^2^ TX = 10 dBm, Interferer = −15 dBm; ^3^ P_in_ = 27.5 dBm, Frequency = 824 MHz; ^4^ TX = 15 dBm, Interferer = −15 dBm.
